# +*Brettanomyces bruxellensis* Displays Variable Susceptibility to Chitosan Treatment in Wine

**DOI:** 10.3389/fmicb.2020.571067

**Published:** 2020-09-04

**Authors:** Margot Paulin, Cécile Miot-Sertier, Lucie Dutilh, Clément Brasselet, Cédric Delattre, Guillaume Pierre, Pascal Dubessay, Philippe Michaud, Thierry Doco, Patricia Ballestra, Warren Albertin, Isabelle Masneuf-Pomarède, Virginie Moine, Joana Coulon, Amélie Vallet-Courbin, Julie Maupeu, Marguerite Dols-Lafargue

**Affiliations:** ^1^EA 4577 OEnologie, INRA, USC 1366, ISVV, Bordeaux INP, Université de Bordeaux, Bordeaux, France; ^2^Microflora-ADERA, EA 4577 OEnologie, ISVV, Bordeaux, France; ^3^CNRS, SIGMA Clermont, Institut Pascal, Université Clermont Auvergne, Clermont-Ferrand, France; ^4^Institut Universitaire de France, Paris, France; ^5^INRA, SupAgro, UM1, UMR 1083, UMR Sciences pour l’Oenologie, Montpellier, France; ^6^Biolaffort, Floirac, France

**Keywords:** chitosan, *Brettanomyces bruxellensis*, antiseptic, wine, spoilage, volatile phenols

## Abstract

*Brettanomyces bruxellensis* is the main spoilage microbial agent in red wines. The use of fungal chitosan has been authorized since 2009 as a curative treatment to eliminate this yeast in conventional wines and in 2018 in organic wines. As this species is known to exhibit great genetic and phenotypic diversity, we examined whether all the strains responded the same way to chitosan treatment. A collection of 53 strains of *B. bruxellensis* was used. In the conditions of the reference test, all were at least temporarily affected by the addition of chitosan to wine, with significant decrease of cultivable population. Some (41%) were very sensitive and no cultivable yeast was detected in wine or lees after 3 days of treatment, while others (13%) were tolerant and, after a slight drop in cultivability, resumed growth between 3 and 10 days and remained able to produce spoilage compounds. There were also many strains with intermediate behavior. The strain behavior was only partially linked to the strain genetic group. This behavior was little modulated by the physiological state of the strain or the dose of chitosan used (within the limits of the authorized doses). On the other hand, for a given strain, the sensitivity to chitosan treatment was modulated by the chitosan used and by the properties of the wine in which the treatment was carried out.

## Introduction

The most feared microbial spoilage in red wines is volatile phenols accumulation, associated with the development of the yeast *Brettanomyces bruxellensis* (Chatonnet et al., 1992; Romano et al., 2009; Comitini et al., 2019). Phenol deviations constitute a criterion for systematic rejection, tarnish the product image and turn buyers away, often permanently. Furthermore, they are perceived not only by professionals but also by consumers, all over the world (Curtin et al., 2007).

The most common method to prevent or eliminate *B. bruxellensis* in wine is sulfur dioxide (SO_2_) addition (Barata et al., 2008). However, the use of SO_2_ can cause undesirable odors of sulfur, hydrogen sulfide formation, and consumption of sulfites causes headaches in many people. As a risk of acute allergy also exists, the European Union has classified SO_2_ as one of the 14 priority food allergens (EU Regulation No. 1169/2011, Annex II). Because the legal dose admitted in both conventional and organic farming will probably be downgraded, but also because of consumer’s expectations, winemakers will need in a near future to reduce the total SO_2_ content in their wines. In addition, massive sulfuring is not always compatible with the production of high quality wines, and it does not always avoid the risk of phenol deviations, because of the emergence of tolerant/resistant *B. bruxellensis* strains (Barata et al., 2008; Agnolucci et al., 2010; Curtin et al., 2012; Longin et al., 2016; Avramova et al., 2018a,b).

Alternate antiseptic molecules or methods are thus needed by winemakers. The resolutions of the 7^*th*^ general assembly of the International Organization of Vine and Wine (International Code of Oenological Practices OIV/OENO, 338A/2009, 2009) and the European Union (EC 53/2011) authorized the use of fungal chitosan for the purpose of reducing undesirable microorganism populations notably *Brettanomyces*, the maximum dose authorized for this purpose being 10 g/hl. OIV recommends subsequent sediments to be removed by physical process, such as racking (International Code of Oenological Practices OIV/OENO, 338A/2009, 2009). The use of fungal chitosan was also recently allowed for organic wine elaboration (Regulation 1584/2018, amending the Regulation EC 889/2008 Annexes).

This antimicrobial solution looks very promising, because fungal chitosan is a highly renewable, no-toxic, non-allergenic biomolecule, which more than 90% is eliminated by racking after wine treatment (Teisseidre et al., 2007). However, fungal chitosan is not used as much as it could because conflicting information is encountered and questions winemaking people: while some advisory sites or publications mention a high antiseptic efficiency for chitosan treatment on wines displaying spoilage microbes, others suggest that it does not always work (Gil et al., 2004; Ferreira et al., 2013; Bagder Elmaci et al., 2015; Taillandier et al., 2015; Petrova et al., 2016).

Several hypotheses could explain the conflicting results reported in the literature and enology advisory websites: first, the batches of chitosan marketed may display heterogeneous quality; secondly, the treated wines may be very different (particularly regarding turbidity and polyphenol content) and thirdly, a great genetic diversity prevails within wine microbial species. This is particularly true for *B. bruxellensis*, the main microbial target of chitosan treatment in wine. *B. bruxellensis* gathers triploid and diploid strains distributed in at least six main genetic groups (Avramova et al., 2018a; Gounot et al., 2020). Wine isolates are also highly diverse and belong to 5 of these genetic groups (Cibrario et al., 2019). This genetic diversity may thus support distinct sensitivities to chitosan as previously described toward sulfites (Curtin et al., 2012; Avramova et al., 2018a,b). In addition, the antimicrobial mechanism of action of chitosan is not entirely deciphered, especially for wine spoilage species (Brasselet et al., 2019).

In this context, our objective was to examine the efficiency of fungal chitosan antiseptic effect on *B. bruxellensis* in wine, taking into account (i) the high genetic diversity of the species, (ii) the possible effects of the fungal chitosan batch quality and (iii) the wine in which the treatment was carried out.

## Materials and Methods

### Microbial Strains

A collection of 53 strains of *B. bruxellensis* was used in this study. These were representative of the six different genetic groups described by Avramova et al. (2018a). Their name, origin and genetic group are indicated in Supplementary Table 1. Thirteen were diploid and belong to the CBS2499 genetic group, 13 were triploid and belong to the genetic group AWRI1499, and 14 were triploid and belong to the genetic group AWRI1608. These three groups are the most frequently encountered in wine (Cibrario et al., 2019). The other 13 strains were distributed into the L14165, L0308, and CBS5512 genetic clusters.

### Chitosan Batches

Two fungal chitosan batches were used, named F1 and F4. Both were sourced by BioLaffort.

### Culture Media and Wines Used

YPD solid medium containing 10 g.L^–1^ yeast extract (Difco Laboratories, Detroit, MI, United States), 10 g.L^–1^ bactopeptone (Difco Laboratories, Detroit, MI, United States), 20 g.L^–1^ D-glucose (Sigma-Aldrich) and 20 g.L^–1^ agar (Sigma-Aldrich) was used for plate counts.

Several wines were used. The first one, wine A, was a “homemade” wine produced in the laboratory from UHT treated commercial red grape juice (a blend from different grape varieties Domaine Laffitte, France), fermented with Zymaflore^®^ F33 (Laffort Oenologie, France). Before storage at 4°C it was filtered (0.4 μm cut-off). The final wine had a pH of 3.57 and contained 12.64% vol ethanol.

The second one, wine B (2016, South France) was a blend of Cabernet Sauvignon and Merlot; It displayed pH 3.56 and 13.19% vol ethanol. The third wine, wine C (2018, Bordeaux area was obtained from Merlot grapes and displayed pH 3.46 and 13.53% vol ethanol).

When specified in the text, the wine A was supplemented with 1 mg/L of *p*-coumaric acid (a metabolic precursor of volatile phenols).

All the wines were treated with H_2_O_2_ to eliminate the total SO_2_, and then pasteurized for 30 min at 80°C, before inoculation and chitosan treatment.

### Chitosan Characterization

#### Viscosity in Solution

A 1% chitosan solution was prepared in 1% acetic acid solution was analyzed with a AR 2000 rheometer, equipped with a Peltier temperature control system (TA-Instrument, AR-2000) and a cone-plane module.

#### Determination of Chitosan Molecular Weight by HPLC/SEC System

Molecular weight (MW) of fungal chitosans F1 and F4 were determined through an HPLC Agilent 1000 series system (Agilent Technologies, United States) with a guard column TSK Gel PW-XL (Tosoh Bioscience, Japan) and two columns TSK Gel 5000 PW-XL and TSK Gel 3000 PW-XL (Tosoh Bioscience, Japan) placed in serial and a refractometric detector (RID). The eluant used was a solution of 0.15M acetic acid and 0.1M sodium acetate filtered degassed through 0.22 μm cellulose filters. Flow rate of the mobile phase was set to 1 mL/min. The column oven was placed at 40°C and a MW calibration curve was done by using pullulans standards (Sigma Aldrich, United States) from 800 to 1.3 kDa. 20 μL of 10 g/L samples and standards were injected into the SEC. Data analysis and control of the HPLC-SEC system is done by Agilent software.

#### Determination of Degree of Acetylation (DA) of Fungal Chitosan by ^1^H-NMR

The ^1^H-NMR spectroscopy analyses were performed on a Bruker Avance DRX 400 spectrometer (Fällanden, Switzerland) in D_2_O-HCl (pH ∼4), at a frequency of resonance of 400 MHz and 80°C. Degree of acetylation (DA), expressed in percentage were estimated according to method largely described in literature (Desbrieres et al., 1996; Lavertu et al., 2003).

#### Residual Glucans Content Determination in Fungal Chitosan Using OIV Standard Method

The residual glucans content in fungal chitosan F1 and F4 was estimated using the method described by OIV Codex resolution (Chitosan monography). In this method we estimated a percentage of residual glucans in fungal chitosan by colorimetric method of Dubois et al. (1956) using the phenol and sulfuric acid assay. Glucose was used as standard.

### Chitosan Treatment of the Inoculated Wines

Chitosan treatment of inoculated wine was performed as recommended by the enological codex. Briefly, the wines were inoculated, mixed with chitosan, let to stand 10 days (the delay recommended by enological codex and by most manufacturers) and then racked.

The strains were gradually adapted to wines before running tests with chitosan. For that, they were first grown in a UHT treated commercial red grape juice at 25° for 5 days and then gradually transferred to pasteurized wine (Cibrario et al., 2020b). The adapted cells generally grew 5 days in the last wine preculture. However, for the experiment exploring the incidence of the yeasts physiological state on chitosan treatment efficiency, the cells grew 3, 7, 10, or 21 days in the last wine preculture. The yeasts were then diluted into wine, in order to ensure an initial population comprised between 10^3^ and 10^5^CFU/mL. A sample was harvested to control the initial population. Then, the inoculated wine was aliquoted into six tubes (13 mL/tube). An aqueous suspension of chitosan F1 (4 g/L) was added to the two first tubes to reach a final concentration of 40 mg/L (i.e., 4 g/hL). The tubes were then gently homogenized by inversion. The same was done to the two following tubes using the second chitosan batch (F4) and the two remaining tubes were kept as controls. Wines were then incubated at 20°C, without any agitation. After 3 days, tubes tubes (one control and a tube treated with each chitosan) were analyzed. The 12 ml up were gently removed and transferred to a fresh tube. This constituted the “racked wine,” while the remaining part of the wine (1 mL left at the bottom of the tube) was considered as lees. Both lees and racked wine were homogenized and analyzed for cultivable population.

After 10 days of incubation at 20°C, the three remaining tubes were analyzed the same way.

For each strain examined, the assay was made twice, using biological duplicate (different *B. bruxellensis* precultures) and a distinct manipulator.

### Cultivable Cells Counts

*Brettanomyces bruxellensis* cultivable populations were measured by serial dilutions in water and plate counts on YPD solid medium. Three plate counts were done for each sample.

### Volatile Phenols Determination

Volatile phenols (4-VP, 4-EP, 4-VG, and 4-EG) were quantified by GC–MS coupled with solid-phase micro-extraction (SPME) on polyacrylate fibers by the method described by Romano et al. (2009). Deuterated 4-ethylphenol (100 μg.L^–1^) was added as an internal standard.

### Calculations and Statistical Analysis

From the populations determined by plate counts, several calculations were realized. We first determined the reduction factor (RF), by comparing the population in the racked wine (after 3 or 10 days of treatment) with the initial population in the wine. RF = initial population/population in the racked wine after treatment.

As a result, considering the detection threshold of the cultivable population determination method (3.3 CFU/mL), for a given assay, the maximal RF was equal to the initial population of the wine divided by 3.3.

Non-parametric Kruskal–Wallis tests were used at α = 5% to identify the means that were significantly different using the agricolae package of the R program (Dray and Dufour, 2007).

## Results

### Chitosan Batches Characterization

Two fungal chitosan batches, F1 and F4, were selected among several others. Both fulfilled the codex requirement regarding insoluble material and tap density (not shown). Note to mention that, both (F1 and F4) displayed low glucan content (<2%) with a total amount of residual glucan estimated at 1.8 and 1.9% for F1 and F4 respectively, despite certified fungal origin. The first one, F1, displayed a low viscosity (<15Cpo) and complied the codex requirements, while the second one, F4, did not comply these requirements (Chitosan monography). However F4 was chosen to examine the importance of the codex requirement for chitosan activity in wine.

^1^H-NMR analysis and HPLC-SEC analysis allowed us to characterize the two fungal chitosan fractions. In fact, as presented in Table 1, F1 displayed a mean MW (estimated by HPLC-SEC) of 32 kDa and a DA (estimated by ^1^H-NMR) of 9.6%, while F4 displayed a mean MW of 400 kDa and a DA of 15.8%. No target DA or MW is indicated in chitosan monography. However, the high MW of F4 may explain its high viscosity in concentrated solution.

**TABLE 1:** Viscosity, molecular weight, and acetylation degree of fungal chitosans F1 and F4.

**Fungal chitosan**	**Viscosity (cPo)**	**M_*W*_ (kDa)**	**DA (%)**
F1	4	32	9.6
F4	113	400	15.8

### Cultivable Populations Evolution Profile During Treatment With Fungal Chitosans

**FIGURE 1:**
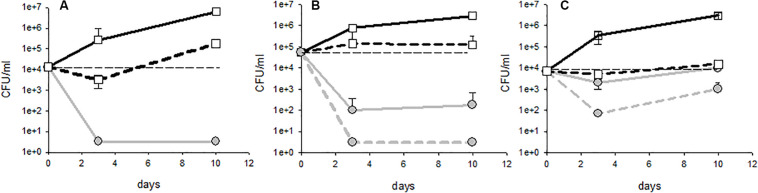
Different profiles of population kinetics observed during treatment with fungal chitosan: **(A)** sensitive strain, **(B)** intermediate profile, and **(C)** tolerant strain. The white squares represent the populations measured in the controls (solid lines: lees; dotted lines: racked wine) and the gray circles represent the populations measured in the test tubes with fungal chitosan (solid lines: lees; dotted lines: racked wine).

The 53 *B. bruxellensis* strains were adapted to wine A in such a way that the yeast populations in the controls tubes were stable or slightly grew during the 10 days of experiment. The initial population varied from 10^3^ to 10^5^ CFU/ml depending on the assay and, we verified that, in the range of initial populations studied and for a given strain, the initial population level did not affect the strain behavior (not shown). In all cases, over the 10 days of experiments, a slight enrichment of the lees compartment (compared to the upper compartment, i.e., the racked wine) was observed, due to yeast cells sedimentation in the control tubes (Figure 1).

Upon treatment with either fungal chitosan F1 or F4, three distinct profiles could be distinguished. First, there were cases in which the populations fell below the detection threshold from day 3, in the racked wine as in the lees (Figure 1A). This concerned 41% of the tests carried out with fungal chitosan F1 and 19% of the tests carried out with fungal chitosan F4. Strains displaying this kind of kinetics were considered “sensitive.” There were then cases where the population in the wine fell close the detection threshold after 3 to 10 days of treatment, but higher populations were detectable in the lees (this concerned 38% of the tests with fungal chitosan F1 and 31% of the tests with fungal chitosan F4, as for example in Figure 1B). These were considered as “intermediate profiles.” Finally, there were assays where populations remained detectable in the racked wine as in the lees and in which we even observed growth between 3 and 10 days (this phenomenon was observed in 13% of the tests with fungal chitosan F1 and 31% with fungal chitosan F4). Strains displaying this profile were considered as “tolerant strains.”

Fungal chitosan thus seems to enhance the sedimentation of the yeasts, but it can also cause cell damage leading to death. This damage would be highly significant in the case of strains with the profile described in Figure 1A (sensitive), and less significant in the case of strains with profile **1B** and **1C** (intermediate and tolerant). These damages were sufficient to prevent the observation of detectable populations of cultivable yeasts at least 1 month after racking in the case of sensitive strains (not shown).

**FIGURE 2:**
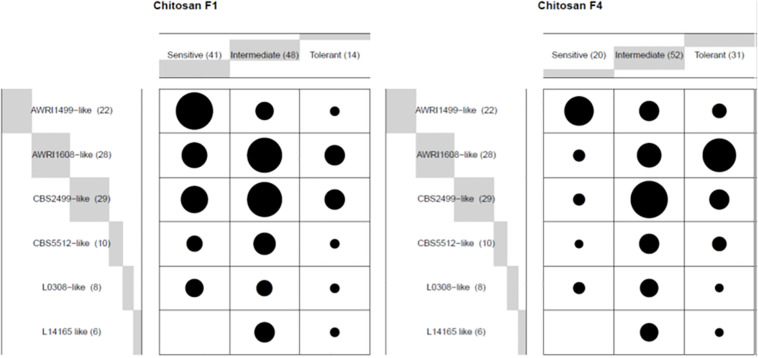
Frequency (ballon plot) of three profiles described in Figure 1, upon treatment with the indicated chitosan batch, for each genetic group in wine A. The circle area is proportional to the number of assays showing the corresponding profile.

We then examined whether the various profiles observed (sensitive, intermediate or tolerant) were evenly distributed into the genetic groups (Figure 2). With chitosan F1, the AWRI1499 genetic group was the one displaying the highest frequency of sensitive behavior, while no sensitive strain was present in group L14165. These groups remained the most sensitive and the less sensitive group respectively with chitosan F4. On the contrary, the proportion of tolerant strains greatly increased in groups AWRI1608 and CBS2499 by changing chitosan F1 by F4. The two remaining groups displayed mainly intermediate strains with F1 as with F4.

### Effectiveness of the Fungal Chitosan Treatment at 10 Days Depending on the Strain Considered

**FIGURE 3:**
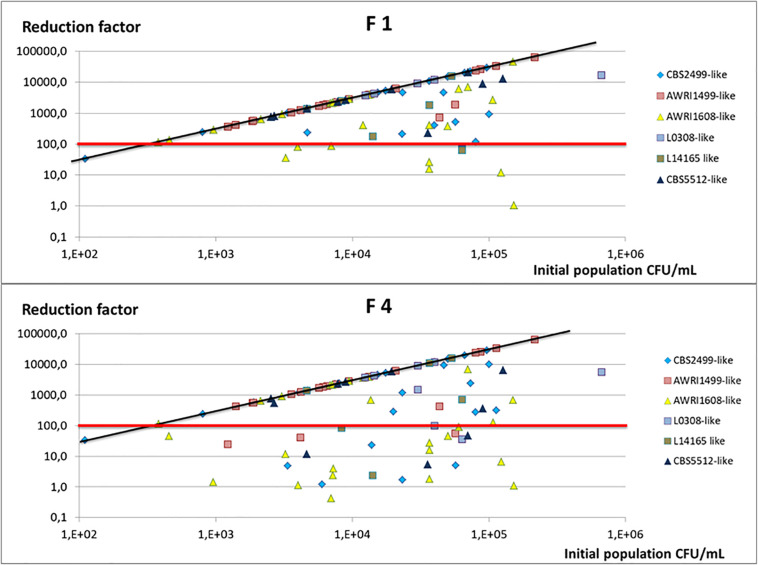
Reduction factor observed in wine A after 10 days of treatment and racking, as a function of the initial population inoculated. Each trial is represented (two points by strain) and the genetic group to which the strain studied belongs is indicated by a color code. The red line indicates the reduction factor 100 (2 log), and the black line the maximal reduction factor expected taking into account the initial population and the population detection threshold (3.33 CFU/mL).

In order to evaluate the chitosan treatment effectiveness from a winemaker’s perspective, we analyzed the initial populations after inoculation of wine A, then the residual populations in the racked wine after 10 days of treatment. From these, the reduction factor was calculated. Figure 3 presents the reduction factors obtained for each trial as a function of the initial population.

Figure 3 makes it possible to compare the reduction factor obtained with the maximum expected one (RFmax = initial population/3.33, 3.33 CFU/mL being the detection threshold of the counting method). With fungal chitosan F1, the reduction factor obtained after 10 days of treatment was greater than 2 LOG in 88% of the cases (dots above the red line) and even equal to the maximum reduction factor for 68% of the trials (dots on the black line). In wine A, the strains of the AWRI1499 genetic group very often displayed a maximum reduction factor with fungal chitosan F1. By opposition, the strains of the other genetic groups were not all equally sensitive to the treatment and, in certain cases, the reduction factor were lower than 100 (dots under the red line).

Concerning experiments using fungal chitosan F4, the treatment was much less effective. The reduction factor was greater than 2 Log in 70% of the assays and was maximal in almost half of the cases (51% of trials). With this chitosan too, strains of the genetic group AWRI1499 also seemed to be the most sensitive, while strains of AWRI1608 genetic group appeared the least sensitive to treatment.

Beyond the reduction factor, what qualifies the effectiveness of the treatment is the level of the final population in the racked wine. For many assays, the population was below or equal to the detection threshold. However, in some cases, even when the reduction factors were satisfactory (>2 log), detectable cultivable populations were still present in the racked wines: in 32% of cases with fugal chitosan F1 (RF < RFmax) and in 49% of cases with fungal chitosan F4. However, the residuals populations were below 10^2^ CFU/mL in 90% of the trials made with chitosan 1 and 75% the trials made with chitosan 4.

**FIGURE 4:**
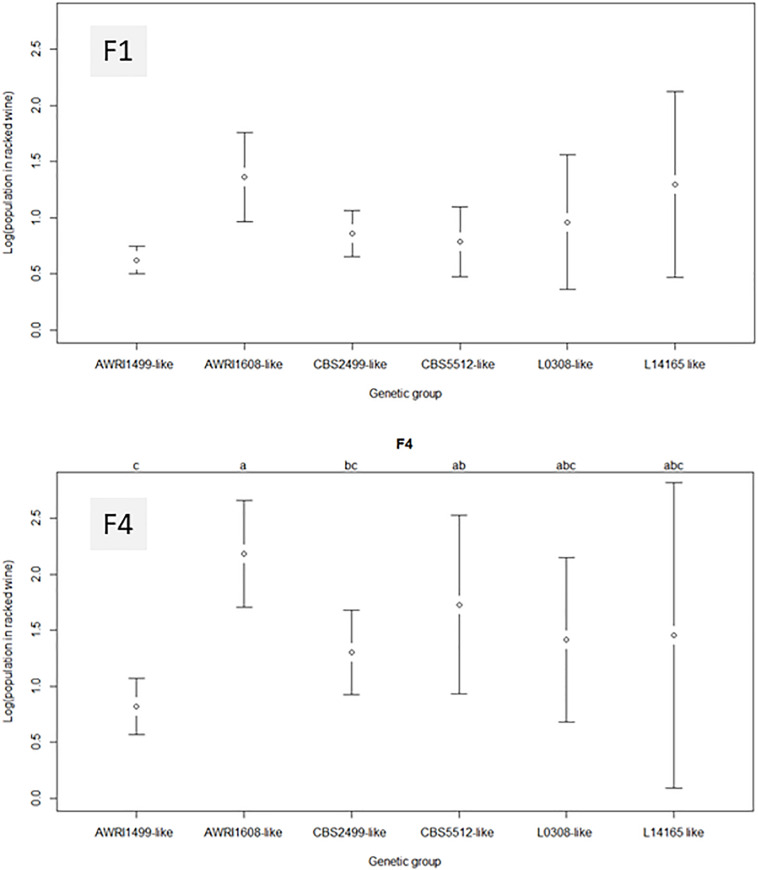
Average residual populations in racked wine for each *B. bruxellensis* genetic group, 10 days after treatment with fungal chitosans F1 or F4. Wine A was used for this study. None of the group studied appeared significantly different from the others with chitosan F1. With F4, the genetic group AWRI1608 was clearly different.

Figure 4 represents the average final population obtained in the racked wine after 10 days of treatment, as a function of the genetic group of the strain and of the fungal chitosan used. In wine A, when the strain present belonged to the genetic group AWRI1499, the final population was close to the detection threshold, regardless of the chitosan used. The *B. bruxellensis* strains in this genetic group seem to be the most affected by chitosan.

With fungal chitosan F1, the average population observed in groups CBS5512 and CBS2499 was also low. However, these two genetic groups gathered strains with more heterogeneous behavior than group AWRI1499. The remaining groups (AWRI1608 L0308 and L14165) appeared even more heterogeneous after treatment. As a result none of the group appeared significantly different from the others in the presence of F1.

With all the groups, the mean residual populations were always higher after treatment with fungal chitosan F4 than after treatment with fungal chitosan F1, which confirmed the lower efficacy of this product compared with the first one. Furthermore, with chitosan F4, the genetic group AWRI1608 displayed residual populations significantly higher than that observed for the other groups, while groups AWRI1499 and CBS2499 displayed significantly lower residual populations.

**FIGURE 5:**
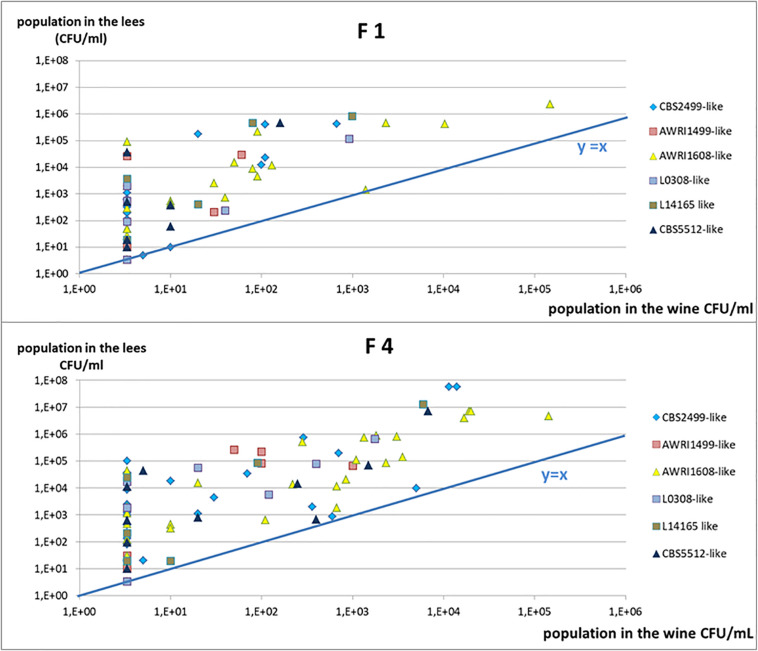
Yeast cultivable population in the lees in function of the populations in the racked wine, after 10 days of treatment. Each trial is represented (two points by strain) and the genetic group to which the strain studied belongs is indicated by a color code.

To better visualize the frequency of the persistence of cultivable yeasts in the lees, we then examined the residual population in the lees as a function of the residual population in the racked wine (Figure 5). Most of the points were found above the curve y = x, in accordance with what it was described in Figure 1: populations in the lees were equal to or higher than that in racked wines, whatever the profile of the strain (sensitive, tolerant, or intermediate). All the tests displaying the profile described in Figure 1A (sensitive strains) were superimposed on the point of coordinate (3.33, 3.33), 3.33 CFU/mL being our detection threshold. All of the tests with intermediate profile (**1B**) give points with abscissa 3.33 and higher ordinate. It thus appears clearly on Figure 5 that, when the racked wine seemed very “clean” (population at or below the detection threshold), the lees could display high cultivable populations, regardless of the batch of fungal chitosan used. Furthermore, for all the trials where detectable populations were present in racked wines, populations in the lees were 10 to 1000 times greater. The strains of the genetic groups AWRI1608 and CBS2499 were most often in this case, whereas the strains of the group AWRI1499 were very often sensitive in wine A.

**FIGURE 6:**
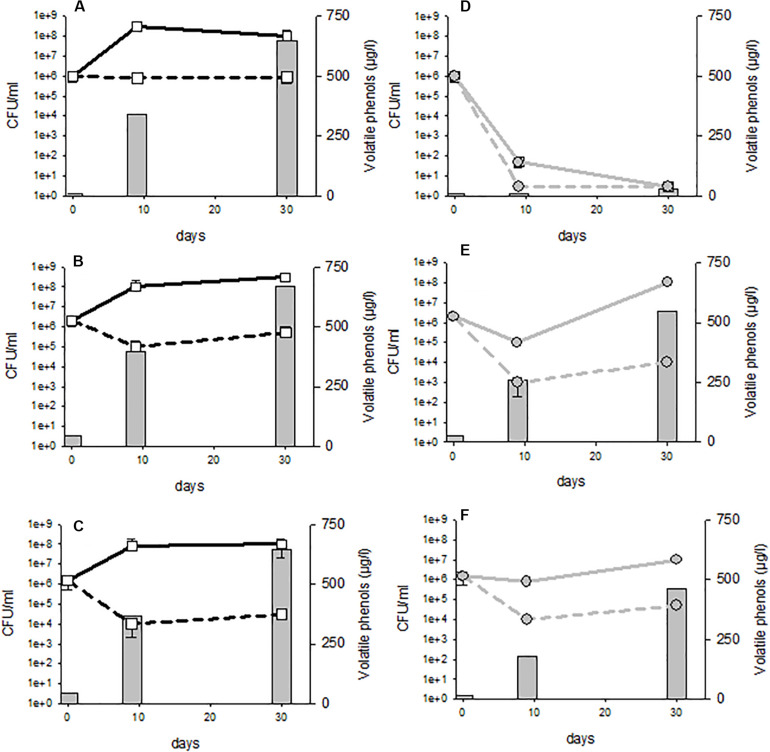
Volatile phenols concentrations and cultivable population evolution in control **(A–C)** or 4 g/hL fungal chitosan F1 treated wines **(D–F)**. Tubes **(A,D)** strain AWRI 1499; **(B,E)** strain CBS2499; **(C,F)** strain AWRI1608.

In order to visualize whether these residual populations in the lees were active and able to produce volatile phenols, the experiment was repeated with three distinct strains belonging to three genetic groups: AWRI1499, AWRI1608 and CBS2499 and displaying different sensitivity profiles. In order to visualize even reduced metabolic activity, the wine was inoculated at high initial population levels (10^6^ CFU/mL) and enriched with ethyl-phenol precursor (*p*-coumaric acid). Samples were withdrawn at 10 and 30 days of treatment and analyzed for cultivable populations and volatile phenol concentrations. The control wines had a perceptible odor deviation from the 10^*th*^ day of experimentation in all cases (Figures 6A–C). The wine alteration was confirmed by chemical analyses: the concentrations of volatile phenols were above the generally accepted olfactory detection threshold of 426 μg/L (Chatonnet et al., 1992). However, none of the trials where fungal chitosan was added displayed noticeable odor after 10 days, and the volatile phenol concentrations were below the olfactory detection threshold, regardless of the chitosan used for the treatment (Figures 6D–F). Although the populations of strains CBS2499 and AWRI1608 were little reduced in treated wines, the treatment, via the reduction of the population in the lees and/or via a direct effect on the metabolic activity of yeasts significantly reduced the rate of phenols accumulation during the 10 first days. The reduction of phenol accumulation remained strong until 30 days with strain AWRI1499 (Figure 6D), and the chitosan treatment seemed very effective. On the other hand, after 30 days, the treated wine containing strains CBS 2499 and AWRI 1608 had a characteristic odor and the phenol concentration was above 426 μg/L (Figures 6E,F). In the case of the CBS2499 strain, this was associated with a significant growth in the lees and in the racked wine (Figure 6E). In the case of the AWRI1608 strain, high phenol production was also observed, while growth was less significant. The cultivable yeasts present in the treated wines are therefore able to produce volatile phenols. Residual populations after treatment are thus potentially dangerous if the treated wine is kept for a long period.

### Influence of the Yeast Physiological State, Fungal Chitosan Dose, and Type of Wine on Microbial Reduction

To assess whether the physiological state of the yeast could modify its sensitivity toward chitosan treatment, we treated wines just after yeast inoculation and after 3, 7, 10, and 21 days of culture. Two strains were studied: a strain classified as sensitive during the first trials (strain L14190, AWRI1499 like, profile **1A**), and a tolerant one (AWRI1608, profile **1C**). The results are presented in Supplementary Figure 1. In all the cases, the controls behaved slightly differently in the oldest cultures: during the 10 days, yeast sedimentation (population decreased in racked wines and enrichment of lees) was more marked than with the “young” inocula, but the whole cultivable populations were maintained. The sensitivity profile observed remained the same: the sensitive strains were sensitive whatever the tests, with populations reduction down to the detection threshold, in wines as in lees from day 3, and the tolerant strain remained tolerant in all cases, with however slightly greater reduction factors with the aged culture (data not shown). In the context of wine A, and within the limits of experience, it seems that the physiological state of a strain does not significantly modify its sensitivity to treatment.

In order to assess whether the fungal chitosan dose modified the strain behavior, experiments were then carried out with these same two strains, in wine A and with different doses of fungal chitosan (0.1; 1, 4, 10, and 100 g/hL). The sensitive strain (L14190) remained sensitive to fungal chitosan F1 even at 1 g/hL, and the tolerant strain remained tolerant, even at the dose of 100 g/hL (Supplementary Figure 2).

**FIGURE 7:**
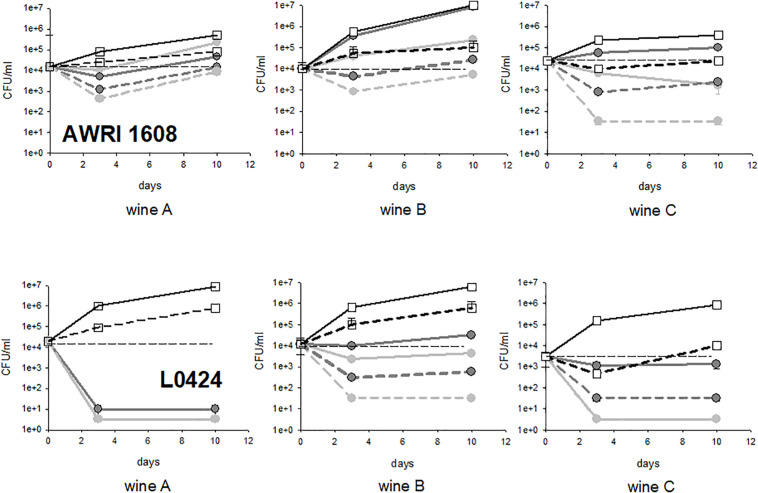
Evolution of cultivable populations of strains AWRI1608 and L0424 in wines A, B, and C treated with fungal chitosans F1 and F4. *The white squares represent the concentrations measured in the control tubes, the light gray circles the tests with fungal chitosan F1 and the dark gray circles those with chitosan F4 (solid lines, lees, dotted lines, racked wine).*

Finally, experiments were performed in three different wines, with strains AWRI 1608 and strain L0424 (AWRI1499 like, sensitive to fungal chitosan). In all the wines examined, the controls had the same type of behavior (steady populations in the wines and growing populations in the lees). Wine A is the homemade wine. Wine B was collected at the end of alcoholic fermentation; it was relatively permissive to *B. bruxellensis* growth, whatever the strain considered. On the contrary, wine C, also collected at the end of alcoholic fermentation, but displaying higher ethanol content and slightly lower pH, was not very permissive to *B. bruxellensis* growth. It required long adaptation steps, except for strain L0424, which is particularly versatile and fitted for growth in all types of wine (Cibrario et al., 2020a,b). The results obtained after treatment with fungal chitosan F1 and F4 are presented in Figure 7. Whatever the wine considered, the strain L0424 was always more sensitive to treatment than strain AWRI1608. However, the very high sensitivity of this strain was observed only in two wines out of 3, with fungal chitosan F1 (wines A and C) and only in one wine among 3 with fungal chitosan F4 (wine A). The strain AWRI1608 was very tolerant in wines A and B and slightly less in wine C, less permissive to its rapid growth.

Wine B was therefore found to be the one in which fungal chitosan treatment was the least efficient as the two strains studied showed significant survival rates in the racked wine as in the lees. Although it is premature to establish a link, this wine was much more loaded with particles and the volume of lees after treatment was very large. Phenomena of inhibition of the action of fungal chitosan by wine particles could be at the origin of the lowest mortality observed in this wine.

Conversely, with the tolerant strain, the treatment was more efficient in wine C than in wines A and B, perhaps through a combined effect of fungal chitosan and other abiotic factors encountered in this wine (alcohol, pH and other inhibitory elements).

## Discussion

All tests combined, fungal chitosan allows reducing *B. bruxellensis* population by a factor higher than 100 in about 80% of cases. Given that, in cellars, the wines to treat generally display initial populations around 10^3^ to 10^4^ CFU/mL, this reduction in population would save a few months of wine stability, if cellar temperatures are well-controlled (Cibrario et al., 2020b). Indeed, when fungal chitosan treatment leads to significant reduction of yeast populations, the volatile phenol production is durably slowed down. However, when populations remain important in the supernatant as in the lees, there may be production of volatile phenols, up to detectable levels, even in the presence of fungal chitosan residues in lees. However, with a small proportion of strains, the treatment is poorly efficient and growth is observed in the presence of fungal chitosan. Furthermore, the lees may present a real danger with measurable cultivable populations in more than 80% of the tests while racked wines did not displayed noticeable cultivable populations in more than 70% of the assays performed. Altogether, the results presented in this study suggest that a final efficient racking is better to eliminate the significant *B. bruxellensis* populations which remain in the lees and that it is not prudent to leave wines in contact with fungal chitosan waste and lees. This should be all the more taken into consideration as the volumes used in this study are small and the tubes shape (conical bottom) facilitate the initial mixture of wine and chitosan and above all, the final separation of wine and lees upon racking.

Two distinct fungal chitosan batches were studied: one obeys all the constraints of the wine codex (fungal chitosan F1), the other not (fungal chitosan F4). The main difference was the average molecular weight, which is much higher in fungal chitosan F4 (400 kDa) inducing then a higher viscosity in media that does not comply with regulations. No target DA is indicated by OIV regulation: F4 displays slightly higher DA and may thus display less positive charge in the wines examined. Our tests clearly indicated that the high molecular weight and less charged fungal chitosan (F4) was less effective for *B. bruxellensis* reduction. However, many studies suggest that a combination of small and larger elements would be necessary for maximum efficiency in wine where racking is performed after treatment. Actually, chitosan causes agglutination and precipitation of the undesired microorganisms and others intracellular events that cause cell death (Helander et al., 2001; Dutta et al., 2012; Zou et al., 2016). The diffusion of low molecular weight chitosan into the cell and its interaction with DNA, RNA, and proteins contribute to cell damage (Liu et al., 2004; Zakrzewska et al., 2005; Eaton et al., 2008; Li et al., 2010; Taillandier et al., 2015). Low and high MW chitosan also contribute together to cell aggregation (No et al., 2002; Zheng and Zhu, 2003; Liu et al., 2005; Mellegård et al., 2011a; Younes et al., 2014). And eventually, high MW insoluble chitosan fractions were shown to act as fining agents, which eliminate cells aggregates (Sudarsan et al., 1992; Strand et al., 2001, 2002; Taillandier et al., 2015). A more precise chemical analysis such as the distribution of *N*-acetyl group versus ^NH_3^+^ function non to the backbone of the fungal chitosans (F1 and F4) could help to further understand the mechanism involved in order to improve the formula of fungal chitosan F1 and propose additional recommendations for fungal chitosan batches quality control, beyond viscosity, glucan content.

Some studies suggest that chitosan mostly causes growth inhibition and that resistant subpopulations might exist (Rhoades and Roller, 2000; Mellegård et al., 2011a,b; Petrova et al., 2016). This is consistent with the behavior observed in the tolerant group. However, the sensitive strains were durably injured, as previously shown by Taillandier et al. (2015). The question is what determines the sensitivity or the tolerant character of a strain? Indeed, we observed partial links between strain sensitivity and genetic group. The AWRI1499 genetic group appeared to be the most sensitive to chitosan while L14165 appears as the less sensitive. The other genetic groups gathered strains with more varied behaviors, with sometimes very high populations in treated wines. The sensitivity of a given strains was little modified by its physiological state or by fungal chitosan dose: it rather appears as a strong and intrinsic characteristic of the strain. Most studies agree that the cationic nature of solubilized chitosan (R-^NH_3^+^ form) interferes with the negatively charged residues of the microbe surface (Rabea et al., 2003; Gomez-Rivas et al., 2004; Raafat et al., 2008). The higher sensitivity of certain strains could therefore be explained by the presence of more charged elements in their cell wall, allowing increased cell binding or entry of chitosan into the cells (Palma Guerreo et al., 2010; Jaime et al., 2012; Tan et al., 2015). These specific elements would be present whatever the stage of development of the yeast and would be specific to or more abundant in certain genetic groups. On the contrary, in the most tolerant groups of strains, surface elements or specific behaviors could help to protect microorganisms (Allan and Hadwiger, 1979; Zakrzewska et al., 2007). Actually, *E. coli* was shown to protect itself by forming aggregates in the presence of chitooligosaccharides (low MW chitosan fractions), which displayed only a bacteriostatic effect leaving the bacteria to grow rapidly after separation from the chitosan by membrane filtration (Eaton et al., 2008; Dutta et al., 2012). This *E. coli* behavior seems similar to that observed with some of the *B. bruxellensis* most tolerant strains in this study.

The group most sensitive to fungal chitosan (AWRI1499) was described to be tolerant to sulfites (Avramova et al., 2018b). Fungal chitosan treatment therefore constitutes a promising solution to lower sulfites concentrations in wines, in case of wine spoilage with strains belonging to this genetic group. However, to predict the effectiveness of fungal chitosan treatment, the identification of the strain present seems not enough, contrarily to what has been developed to predict the effectiveness of SO_2_ treatment (Avramova et al., 2018b). The wine context also must be taken into account, as fungal chitosan treatment appeared less effective in certain wines and reminds the differences of chitosan treatment efficiency reported between laboratory media and foods (Rhoades and Roller, 2000; Kiskó et al., 2005; Hosseinnejad and Jafari, 2016). In each wine, superimposition of two phenomena may contribute to chitosan final efficiency for a given strain present: a masking of chitosan active fraction by steric hindrance or linking to wine compounds, which may decrease chitosan efficiency, and a direct effect of the wine composition and characteristics (pH, alcohol, and others) on yeast growth inhibition, which may indirectly (unless synergistic effect exist) contribute to higher chitosan efficiency.

## Conclusion

This is the first study to report the characterization of chitosan impact on *B. bruxellensis* that takes into account the genetic factor (53 tested strains representative of the great genetic diversity of the species), the impact of the wine matrix (three red wines) and the impact of the chitosan batch (two batches). All the 53 *B. bruxellensis* strains tested in this study were affected (at least transiently): fungal chitosan is a broad-spectrum antiseptic agent. Nevertheless, in the conditions tested, it is not 100% efficient as cultivable population is detected in about 30% of the assay performed overall the present study. Actually, fungal chitosan F1 and F4 are not equal and all the strains are not as sensitive. The comparison of different fungal chitosan preparations, further analysis of the physiological consequences of treatment or the comparison of the biochemical and genetic properties of resistant and sensitive strains will help us to better understand fungal chitosan mechanism of action, to better control its activity. Furthermore, work will be necessary in order to identify the elements of the wine or the stages of winemaking unfavorable to the treatment with fungal chitosan. Research is also needed to state about the durability of the treatment and to examine its consequences on wine sensorial properties, before providing new advices for a better use of fungal chitosan treatment.

## Data Availability Statement

The raw data supporting the conclusions of this article will be made available by the authors, without undue reservation.

## Author Contributions

MD-L, CD, JM, JC, and TD designed the experiment. MP, CM-S, LD, CB, PD, and GP realized the experiments. MP, CM-S, and WA performed the statistical analysis. WA, PB, IM-P, VM, JC, PM, CD, AV-C, JM, TD, and MD-L wrote and carefully read the manuscript. All authors contributed to the article and approved the submitted version.

## Conflict of Interest

LD, AV-C, and JM were employed by Microflora-ADERA and JC and VM by Biolaffort. The remaining authors declare that the research was conducted in the absence of any commercial or financial relationships that could be construed as a potential conflict of interest.
