# Supplementary material for: +Brettanomyces bruxellensis Displays Variable Susceptibility to Chitosan Treatment in Wine
Source: Front Microbiol. 2020 Sep 4;11:571067. doi: 10.3389/fmicb.2020.571067 (PMC7498638; doi:10.3389/fmicb.2020.571067)
Supplement: Supplementary file 1 [file Data_Sheet_1.PDF]

Supplemental table 1.

Strain	Origin	Genetic group
AWRI1499	wine	
ISA2404	wine	
L1792	Kombucha	
L1793	Kombucha	
L0417*	wine	
L0424*	wine	
L0516*	wine	AWRI1499
L06/034AZ	wine	
L14156*	wine	
L14190*	wine	
LB15109p*	wine	
UWOPS94-239.3	Tequila	
VP1545	wine	
13EN11C5	wine	
11AVB4	wine	
CBS 2499	wine	
DEN612_9	wine	
L02/E2 AZ	wine	
L0469*	wine	
L0611*	wine	CBS2499
L14160*	wine	
L14163*	wine	
L14168*	wine	
L14186*	cider	
CBS 6055	beer	
L17105	Kombucha	
AWRI1608	wine	
AWRI1677	wine	
CDR202	wine	
GSP1504	beer	
GSP1516	beer	
GSP1518	beer	
ISA1700	wine	AWRI1608
L0422*	wine	
L0463*	wine	
L14173*	wine	
L14183*	wine	
L14195*	beer	
YJS5400	wine	
L1120*	white wine	
L1710*	wine	
L0308*	wine	
L1757*	unknown	L0308
L1781*	wine	
B001-14 T28 1 bis	wine	
L17103	Kombucha	
L17104	Kombucha	L14165
CBS3025 bis	beer	
UWOPS92-244.4	Tequila	
UWOPS92-255.4	Tequila	
UWOPS92-262.3	Tequila	CBS5512
JP258V2013.C7	Bioethanol	
JP354V2014.C8	Bioethanol	
