# Supplementary material for: +Brettanomyces bruxellensis Displays Variable Susceptibility to Chitosan Treatment in Wine
Source: Front Microbiol. 2020 Sep 4;11:571067. doi: 10.3389/fmicb.2020.571067 (PMC7498638; doi:10.3389/fmicb.2020.571067)
Supplement: Supplementary file 2 [file Data_Sheet_2.PDF]

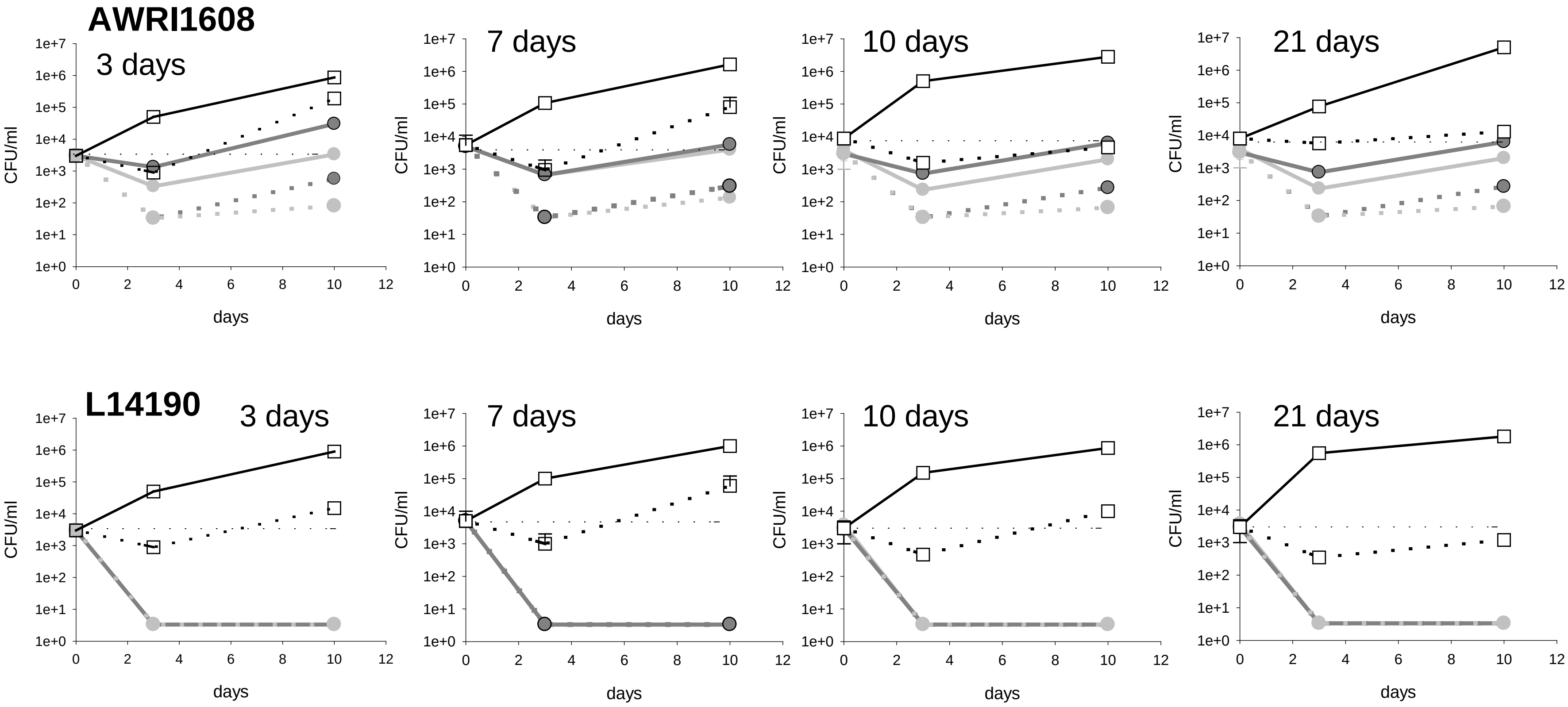

Supplemental figure 1

Evolution of cultivable populations of strains AWRI1608 and L0424 in wine A ,inoculated with 3, 7 ,10 or 21 days old preculture, and treated with fungal chitosans F1 and F4. The white squares represent the concentrations measured in the control tubes, the light gray circles the tests with fungal chitosan F1 and the dark gray circles those with chitosan F4 (solid lines, lees, dotted lines, racked wine).
