# Supplementary material for: +Brettanomyces bruxellensis Displays Variable Susceptibility to Chitosan Treatment in Wine
Source: Front Microbiol. 2020 Sep 4;11:571067. doi: 10.3389/fmicb.2020.571067 (PMC7498638; doi:10.3389/fmicb.2020.571067)
Supplement: Supplementary file 3 [file Data_Sheet_3.PDF]

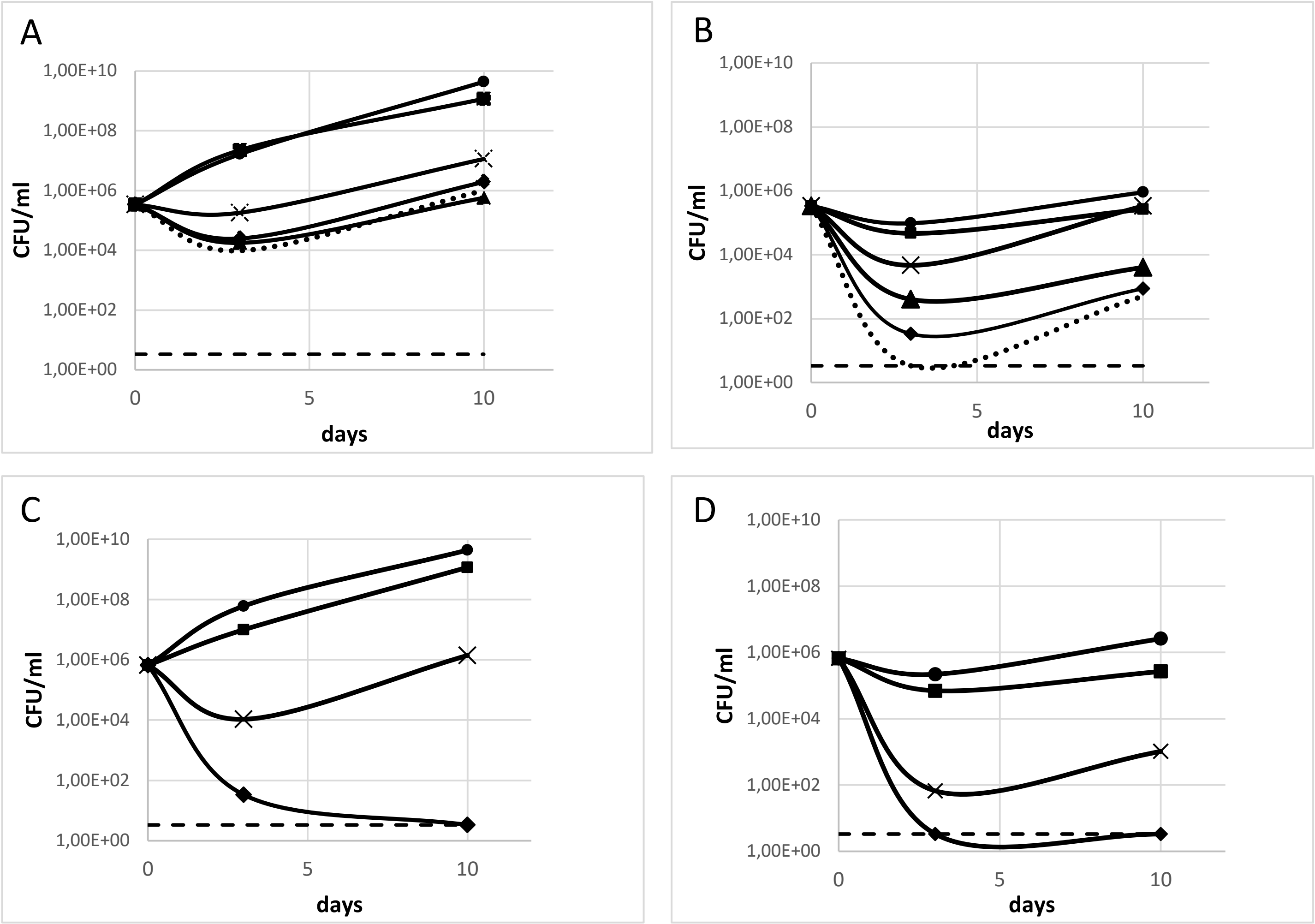

Supplemental figure 2: Evolution of cultivable population of strains AWRI 1608 and L 14190 in wine A, treated with graduated doses of fungal chitosan F1. A) Strain AWRI 1608 cultivable population in lees B) Strain AWRI 1608 cultivable population in racked wine C) Strain L14190 cultivable population in lees D) L14190 cultivable populations in racked wine.

(●) represent non treated population. (■), (✖) and (◆) show respectively population after treatment with 0,01g/hL, 0,1g/hL and 1g/hL of chitosan. (▲) and (······) represent the population after treatmenrt with 10g/hL and 100g/hL of chitosan. The dotted line represent the detection threshold.
